# Preoperative positive voided urine cytology predicts poor clinical outcomes in patients with upper tract urothelial carcinoma undergoing nephroureterectomy

**DOI:** 10.1186/s12885-020-07623-5

**Published:** 2020-11-16

**Authors:** Wen Liu, Zhankun Wang, Shuai Liu, Yu Yao, Yong Liu, Guiming Zhang

**Affiliations:** 1 Department of Urology, The Affiliated Hospital of Qingdao University, No. 16, Jiangsu Rd, Qingdao, 266003 China; 2 Department of Urology, Qingdao Eighth People’s Hospital, Qingdao, China

**Keywords:** Upper urinary tract, Urothelial carcinoma, Nephroureterectomy, Recurrence, Survival, Urine cytology

## Abstract

**Background:**

Performance of urinary cytology is recommended as the part of a standard diagnostic workup and base surveillance regimens in upper tract urothelial carcinoma (UTUC). However, the effect of positive voided urine cytology (VUC) on UTUC prognosis, compared with negative VUC, has not been fully demonstrated. This study aimed to evaluate the impact of preoperative VUC on predicting intravesical recurrence, disease recurrence, and mortality in patients with UTUC who underwent nephroureterectomy (RNU).

**Methods:**

Clinicopathological information was collected from 315 UTUC patients treated with RNU. The association between VUC and oncological outcomes was analyzed using the Kaplan–Meier method with log-rank test and Cox proportional hazards regression models. Multiple logistic regression analysis was performed to identify the influence of VUC on tumor grade.

**Results:**

Preoperative positive VUC, presenting in 101 patients (32%), was significantly associated with tumor multifocality (*P* = 0.017) and higher tumor grade (*P* = 0.010). On multivariable Cox regression analyses, preoperative positive VUC was an independent prognostic factor of intravesical recurrence-free survival (RFS) (hazard ratio [HR] = 2.21, 95% confidence interval [CI] 1.06–4.64; *P* = 0.035), RFS (HR = 1.80, 95% CI 1.08–2.99; *P* = 0.023), and cancer-specific survival (CSS) (HR = 1.87, 95% CI 1.10–3.18; *P* = 0.020), but not overall survival (HR = 1.32, 95% CI 0.80–2.18; *P* = 0.28). Logistic regression analysis revealed that VUC was related to high tumor grade in UTUC (odds ratio = 2.23, 95%CI 1.15–4.52).

**Conclusion:**

Preoperative positive VUC significantly increases the risk of intravesical recurrence in UTUC patients undergoing RNU. In addition, positive VUC is an adverse predictor of RFS and CSS, which might be due to the association between positive VUC and high tumor grade.

## Background

Upper urinary tract urothelial carcinoma (UTUC) is relatively uncommon and accounts for only 5.0–10% of all urothelial carcinomas [1]. Radical nephroureterectomy (RNU) with bladder cuff excision is the surgical standard of care for UTUC [2]. However, the 5-year cancer-specific survival (CSS) remains < 50% for pT2/pT3 and < 10% for pT4 after surgery [1]. Although cisplatin-based adjuvant chemotherapy has a demonstrated survival benefit in UTUC [3], identification of prognostic indicators and risk stratification are important for developing appropriate follow-up regimens and selecting suitable adjuvant therapies.

Performance of urinary cytology is recommended as part of the standard diagnostic work-up and base surveillance regimens in UTUC. Furthermore, in comparison with low-grade urine cytology, high-grade cytology is considered a high-risk prognostic factor in UTUC patients [1]. However, the effect of positive voided urine cytology (VUC) on UTUC prognosis compared with negative VUC has not been fully demonstrated. Several studies suggested that preoperative positive VUC increased the risk of bladder recurrence after RNU; however, the results remain controversial [4–9]. The underlying mechanisms for subsequent bladder tumors might be intraluminal seeding or intraepithelial spread of cancer cells and field cancerization [5]. In addition, Sakano et al. found that preoperative VUC increased cancer-specific mortality in UTUC [10], which might indicate that preoperative VUC is a predictive tool for high-grade or invasive UTUC [11–13]. Therefore, we aimed to fully investigate the impact of preoperative VUC for predicting oncological outcomes in UTUC following RNU, including intravesical recurrence, disease recurrence, and mortality.

## Methods

### Patient selection

A total of consecutive 341 UTUC patients who retained VUC and underwent RNU between January 2012 and April 2019 at the Department of Urology, The Affiliated Hospital of Qingdao University were enrolled in this study. RNU was performed via an open or laparoscopic approach. Regional lymphadenectomy was routinely performed in patients with suspected enlarged nodes in preoperative computed tomography (CT) or intraoperative examinations. We used cystoscopy before or during RNU to exclude the possibility of concomitant bladder cancer. Of the 341 patients, 26 were excluded for the following reasons: bladder cancer before and/or during RNU (*n* = 10), other cancers before, during, and/or after RNU (*n* = 9), distant metastasis (*n* = 4), and pathological lymph node metastases (*n* = 3). None of the enrolled patients accompanied contralateral UTUC before RNU. Finally, 315 UTUC patients (pTa-4N0M0) without neoadjuvant therapy were enrolled in this study. The study protocol was approved by the ethics committee at our institution. All patients involved in the present study provided signed informed consent.

### Evaluation of variables

Genitourinary pathologists at our institution evaluated the voided urine samples. Negative VUC was defined as a negative result and/or a report of atypical cells, while positive VUC was defined as a positive result and/or a suspicious report [8].

Tumors were staged according to the 2018 American Joint Committee on Cancer (AJCC) TNM staging system and graded according to the 2004 World Health Organization grading system [14]. Tumor multifocality was defined as a total of ≥2 pathologically confirmed tumors in the renal pelvis and/or ureter. The size of multifocal tumors equaled the diameter of the dominant lesion. Gross hematuria or microscopic hematuria (three or more red blood cells per high-powered field) before surgery was considered hematuria [15]. Lymphovascular invasion (LVI) is defined pathologically as the presence of tumor cells within an endothelium-lined space without underlying muscular walls [16]. We considered the application of intravesical chemotherapy (epirubicin, mitomycin C, and pirarubicin) after RNU as a positive history of bladder instillation. Other clinicopathological data included sex, age, smoking and drinking history, history of hypertension and diabetes, body mass index, ipsilateral hydronephrosis, preoperative ureteroscopy (URS), and tumor location (renal pelvis, ureter, or both).

### Follow-up protocol

Patients were followed up with cystoscopic examinations every 3 months for 2 years after RNU, then every 6 months from 3 to 5 years, and annually after 5 years. Urine cytology, CT and/or magnetic resonance imaging was performed annually to detect bladder or local recurrence and distal metastasis. We defined intravesical recurrence as pathologically confirmed bladder urothelial carcinoma after RNU. Disease recurrence was considered as local recurrence, lymph node invasion, and/or distant metastasis, except for contralateral upper urinary tract or subsequent bladder recurrence. Cause of death was determined by death certificates and results were estimated by treating physicians.

### Statistical analyses

The correlation between preoperative VUC and the other categorical variables was tested by chi-squared test. Survival curves were estimated by the Kaplan–Meier method and compared using the log-rank test. The Cox proportional hazards regression models were used in univariable and multivariable regression analyses to evaluate the effect of VUC on UTUC survival. We added an interaction term, consisting of VUC and tumor grade, to the Cox multivariable analyses to test the interaction between VUC and tumor grade. Multiple logistic regression analysis was performed to identify the impact of VUC on tumor grade. Statistical analyses were performed using R software (R 3.5.1) and SPSS (V. 24.0). All reported *P*-values were two-sided, and statistical significance was set at *P* < 0.05.

## Results

A total of 315 patients who had undergone RNU were included in this study, and consisted of 192 (61%) males and 123 (39%) females. They were diagnosed with UTUC at a median age of 67 years (interquartile range, 61–75 years). The median overall follow-up period was 32 months (interquartile range, 14–48 months), the median follow-up duration of patients alive and disease-free was 29 months. The distributions of clinicopathological characteristics are shown in Table 1. Positive VUC, present in 101 patients (32%), was significantly associated with tumor multifocality (*P* = 0.017) and higher tumor grade (*P* = 0.010) (Table 1).

**Table 1:** Association of voided urine cytology with clinicopathological characteristics in UTUC patients

	Urine Cytology		
	Negative (%)	Positive (%)	*P* value
Gender			
Male	126 (41.1)	66 (34.7)	0.27
Female	88 (58.9)	35 (65.3)	
Age (years)			
<65	88 (41.1)	33 (32.4)	0.15
≥ 65	126 (58.9)	68 (67.3)	
Smoking history			
No	141 (65.9)	64 (63.4)	0.7
Yes	73 (34.1)	37 (36.6)	
Alcohol history			
No	164 (76.6)	70 (69.3)	0.17
Yes	50 (23.4)	31 (30.7)	
Hematuria			
No	45 (21.0)	8 (7.9)	0.004
Yes	169 (79.0)	93 (92.1)	
Hypertension			
No	139 (65.0)	55 (54.5)	0.074
Yes	75 (35.0)	46 (45.5)	
Diabetes mellitus			
No	182 (85.0)	84 (83.2)	0.7
Yes	32 (15)	17 (16.8)	
Body mass index			
<30	194 (90.7)	93 (92.1)	0.7
≥ 30	20 (9.3)	8 (7.9)	
Hydronephrosis			
No	76 (35.5)	40 (39.6)	0.5
Yes	138 (64.5)	61 (60.4)	
Multifocality			
Unifocal	200 (93.5)	86 (85.1)	0.017
Multifocal	14 (6.5)	15 (14.9)	
Size			
≤ 2 CM	59 (27.6)	25 (24.8)	0.6
>2 CM	155 (72.4)	76 (75.2)	
Bladder instillation			
No	49 (22.9)	26 (25.7)	0.6
Yes	165 (77.1)	75 (74.3)	
Pathologic tumor stage			
≤ T2	114 (53.3)	45 (44.6)	0.15
≥ T3	100 (46.7)	56 (55.4)	
Tumor grade			
Low grade	61 (29.2)	15 (15.5)	0.010
High grade	148 (70.8)	82 (84.5)	
Location			
Renal pelvis	101 (48.1)	54 (57.4)	0.13
Ureter	109 (51.9)	40 (42.6)	
Preoperative URS			
No	160 (74.8)	84 (83.2)	0.10
Yes	54 (25.2)	17 (16.8)	
LVI			
No	62 (59.0)	35 (64.8)	0.5
Yes	43 (41.0)	19 (35.2)	

*LVI* Lymphovascular invasion; *CM* Centimeter; *URS* Ureteroscopy

**Fig. 1:**
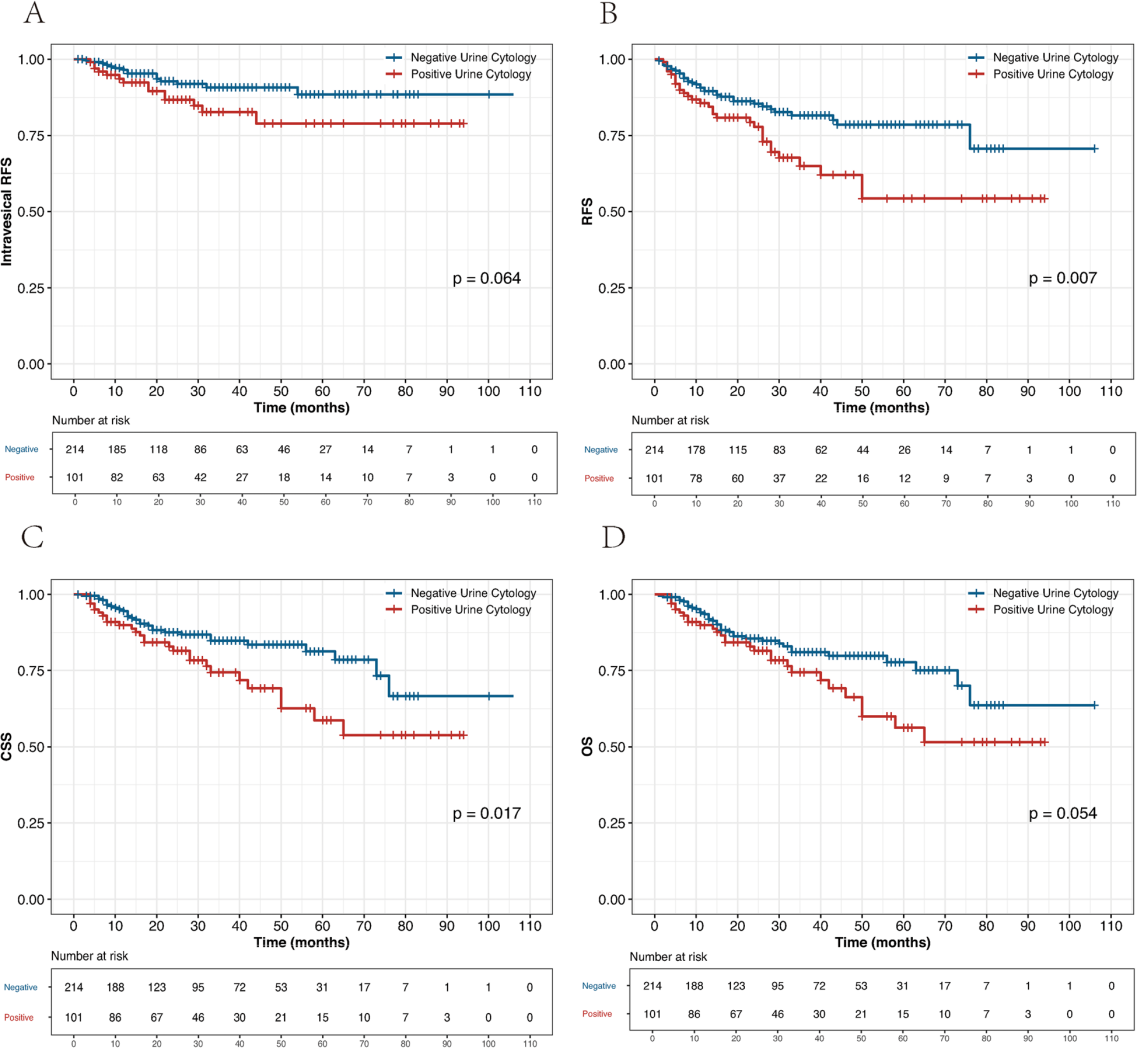
Kaplan–Meier curves for intravesical RFS (**a**), RFS (**b**), CSS (**c**), and OS (**d**) stratified by preoperative voided urine cytology in 315 UTUC patients

During the follow-up, 29 patients (9.2%) experienced pathological bladder recurrence, among which the number of non-muscle-invasive bladder cancer (NMIBC) (pT ≤ T1) and high-grade tumors were 23 (79%) and 22 (76%), respectively. NMIBC was treated by transurethral resection of the bladder (TURB) in combination with intravesical instillations. Four patients with muscle-invasive bladder cancer (MIBC) underwent radical cystectomy, one patient with MIBC underwent partial cystectomy, and one patient with MIBC underwent pelvic chemotherapy. Sixty-four patients (20%) experienced disease recurrence and 57 patients (18%) died of UTUC. A total of 7 people died of other causes, of which 3 died of stroke, 2 died of lung infection, 1 died of myocardial infarction, and 1 died of trauma. Five-year intravesical recurrence-free survival (RFS), RFS, CSS, and overall survival (OS) rates for the positive and negative VUC groups were 79% vs. 89% (*P* = 0.064; Fig. 1a), 70% vs. 79% (*P* = 0.007; Fig. 1b), 57% vs. 81% (*P* = 0.017; Fig. 1c), and 56% vs. 78% (*P* = 0.054; Fig. 1d), respectively.

**Fig. 2:**
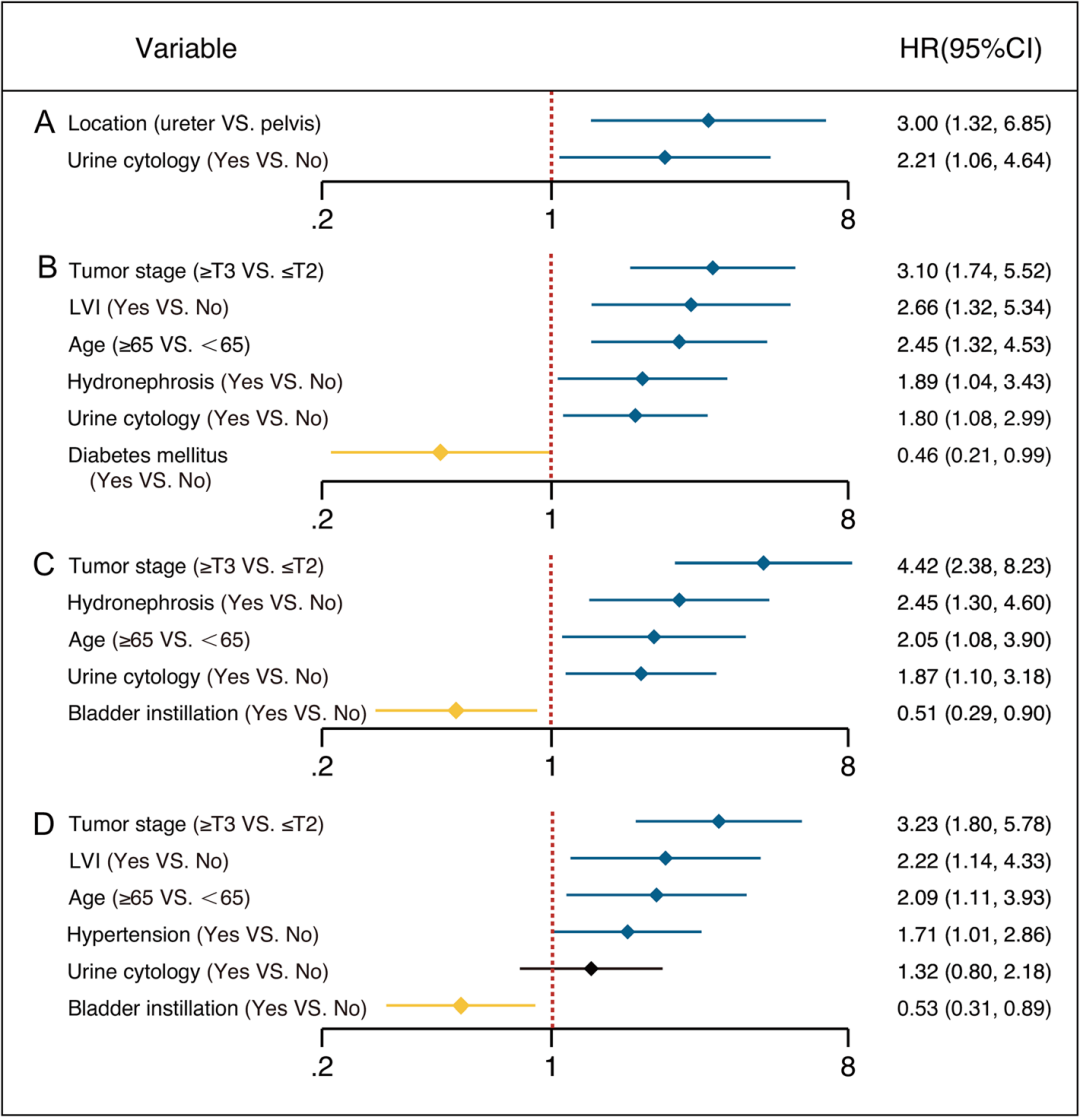
Positive outcomes of multivariable Cox regression models for intravesical RFS (**a**), RFS (**b**), CSS (**c**), and OS (**d**)

In multivariable Cox regression analyses, tumor location and positive preoperative VUC were prognostic indicators for intravesical recurrence (Fig. 2a). LVI, higher pT stage (≥pT3), advanced age, preoperative hydronephrosis, and positive VUC increased the risk of disease recurrence in UTUC patients (Fig. 2b). Tumor stage, hydronephrosis, age, positive VUC, and bladder instillation predicted cancer-specific death after RNU (Fig. 2c). Independent predictors of OS were tumor stage, LVI, age, and bladder instillation (Fig. 2d). Multivariable analyses revealed that positive preoperative VUC was significantly associated with poor intravesical RFS (hazard ratio [HR] = 2.21, 95% confidence interval [CI] 1.06–4.64; *P* = 0.035), RFS (HR = 1.80, 95% CI 1.08–2.99; *P* = 0.023), and CSS (HR = 1.87, 95% CI 1.10–3.18; *P* = 0.020), but not OS (HR = 1.32, 95% CI 0.80–2.18; *P* = 0.28) (Table 2). The interaction term addressing the combination of VUC and pathologic tumor grade on RFS, CSS, and OS failed to reach statistical significance (*p* = 0.35, 0.23, and 0.26, data not shown), indicating that the risk associated with the presence of VUC and tumor grade did not appear to exceed the additive contribution of these risk variables.

**Table 2:** Univariable and multivariable Cox regression analyses of all patients for intravesical recurrence-free survival, recurrence-free survival, cancer-specific survival, and overall survival

Factors	Intravesical RFS			RFS			CSS			OS		
	Univariable	Multivariable^a^		Univariable	Multivariable^b^		Univariable	Multivariable^c^		Univariable	Multivariable^c^	
	*P*	HR(95%CI)	*P*	*P*	HR(95%CI)	*P*	*P*	HR(95%CI)	*P*	*P*	HR(95%CI)	*P*
**Urine cytology (Yes VS. No)**	**0.073**	**2.21 (1.06–4.64)**	**0.035**	**0.009**	**1.80 (1.08–2.99)**	**0.023**	**0.019**	**1.87 (1.10–3.18)**	**0.020**	**0.057**	**1.32 (0.80–2.18)**	**0.28**
Tumor stage (≥T3 VS. ≤T2)	0.5		0.28	<0.001	3.10 (1.74–5.52)	<0.001	<0.001	4.42 (2.38–8.23)	<0.001	<0.001	3.23 (1.80–5.78)	<0.001
Age (≥65 VS. <65)	0.3		0.6	0.001	2.45 (1.32–4.53)	0.004	0.002	2.05 (1.08–3.90)	0.029	0.002	2.09 (1.11–3.93)	0.022
Bladder irrigation (Yes VS. No)	0.25		0.15	0.20		0.25	0.050	0.51 (0.29–0.90)	0.021	0.024	0.53 (0.31–0.89)	0.017
LVI (Yes VS. No)	0.19		0.051	<0.001	2.66 (1.32–5.34)	0.006	<0.001		0.076	0.003	2.22 (1.14–4.33)	0.019
Hydronephrosis (Yes VS. No)	0.13		0.8	0.061	1.89 (1.04–3.43)	0.036	0.094	2.45 (1.30–4.60)	0.005	0.23		0.17
DM (Yes VS. No)	0.7		–	0.10	0.46 (0.21–0.99)	0.047	0.6		–	0.6		–
Location (ureter VS. pelvis)	0.028	3.00 (1.32–6.85)	0.009	0.21		0.4	0.23		0.4	0.8		0.3
Hypertension (Yes VS. No)	0.26		–	0.049		0.10	0.044		0.13	0.022	1.71 (1.01–2.86)	0.044
Gender (Male VS. Female)	0.7		–	0.8		–	0.6		0.5	0.21		0.091
Smoking (Yes VS. No)	0.6		0.8	0.5		0.4	0.6		0.6	0.9		0.8
BMI (≥30 VS. <30)	0.5		0.4	0.18		0.4	0.3		0.6	0.23		0.4
Multifocality (Yes VS. No)	0.4		0.6	0.034		0.13	0.12		0.29	0.22		0.7
Size (>2 CM VS. ≤2 CM)	0.7		0.3	0.9		0.4	1		0.8	1		0.9
Tumor grade (high VS. low)	0.4		0.8	0.014		0.11	0.018		0.063	0.027		0.23
Hematuria (Yes VS. No)	0.034		0.054	0.7		–	0.6		–	0.4		–
Alcohol history (Yes VS. No)	0.050		0.073	0.9		–	1		–	0.5		–
Preoperative URS	0.20		0.6	–		–	–		–	–		–

Note: ^a^ Adjusted for tumor stage, age, bladder irrigation, LVI, hydronephrosis, location, smoking, BMI, multifocality, size, tumor grade, hematuria, alcohol history, and URS;

^b^ Adjusted for tumor stage, age, bladder irrigation, LVI, hydronephrosis, DM, location, hypertension, smoking, BMI, multifocality, size, and tumor grade;

^c^ Adjusted for tumor stage, age, bladder irrigation, LVI, hydronephrosis, location, hypertension, gender, smoking, BMI, multifocality, size, and tumor grade;

Abbreviations: *RFS* Recurrence-free survival, *CSS* Cancer-specific survival; *OS* Overall survival, *DM* Diabetes mellitus; *BMI* Body mass index; *LVI* Lymphovascular invasion; *URS* Ureteroscopy, *HR* Hazard ratio

**Fig. 3:**
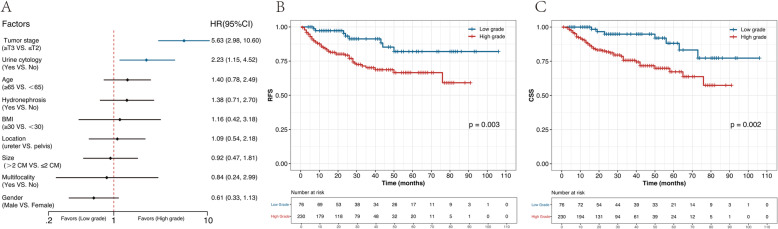
To investigate the risk factors for high-grade UTUC by multivariable logistic regression analysis (**a**), Kaplan–Meier curves for RFS (**b**), and CSS (**c**) were stratified by tumor grade in UTUC patients

In multiple logistic regression analysis, preoperative VUC was an independent predictor of histological high tumor grade in UTUC (odds ratio [OR] = 2.23, 95% CI 1.15–4.52) (Fig. 3a). Log-rank test verified that higher grade was significantly associated with poor RFS (*P* = 0.003) and CSS (*P* = 0.002) (Fig. 3b, c).

## Discussion

In the present study, we found that preoperative VUC was associated with high tumor grade, which indicated biologically aggressive UTUC. In addition, preoperative positive VUC significantly increased the risk of intravesical recurrence. We confirmed that VUC before RNU was an independent prognostic factor for disease recurrence and cancer-specific mortality, but not overall mortality.

In agreement with several previous studies [5, 8, 9], we found that preoperative VUC was an independent predictor of bladder recurrence in UTUC patients (HR = 2.21, 95% CI 1.06–4.64; *P* = 0.035). Currently, two hypotheses have been proposed to explain bladder recurrence following RNU [5]. One is field cancerization, in which exposure to carcinogens throughout the entire urothelium leads to independent multifocal tumor development [17]. A more important view is intraluminal seeding and implantation of cancer cells from the upper urinary tract to the bladder [18]. In favor of the intraluminal seeding theory, studies reported that the intravesical recurrence rate in patients undergoing surgery for UTUC was 22–47% [18], while UTUC prevalence after cystectomy ranged from 0.75 to 6.4% [19]. The occurrence of contralateral UTUC was much less than that of intravesical recurrence after RNU, ranging from 2 to 6% [1]. Moreover, a meta-analysis proved that ureteroscopy before RNU did not improve RFS, CSS, and OS in UTUC patients, but increased the risk of intravesical recurrence. It may be that ureteroscopy promotes the implantation of tumor cells isolated from UTUC into the bladder [20]. Therefore, it is reasonable that through seeding and planting of cancer cells, positive preoperative VUC significantly increased the risk of bladder recurrence after RNU. A multi-institutional study reported that positive preoperative urine cytology was a risk factor for intravesical recurrence after RNU, and early ligation of the ureter distal during surgery could not reduce the risk of intravesical recurrence after RNU [9]. Thus, they hypothesized that cancer cells continuously detach from UTUC to bladder in the preoperative period leading to bladder recurrence [9]. Akihiro et al. found that most sites of bladder recurrence were located in likely injured urothelium, which could serve as a site for tumor cell adhesion [5]. Furthermore, the present study revealed that, compared with the renal pelvis, UTUC in the ureter was associated with bladder recurrence after RNU, indicating that the anatomic proximity to the bladder might largely promote intravesical implantation of tumor cells [18].

Intravenous chemotherapy after RNU for UTUC had no effect on intravesical RFS (*P* = 0.15) in this study but improved the outcome of CSS (*P* = 0.021) and OS (*P* = 0.017) (Table 2). Previous studies examined different chemotherapy drugs and bladder instillation regimens, yielding different results [21]. Regardless of the intravesical chemotherapy protocol, we grouped all individuals who received intravesical chemotherapy after RNU into those with a positive history of bladder instillation, which might weaken the effect of intravesical chemotherapy on UTUC. Based on the intraluminal seeding hypothesis, we believe that it is rational to administer postoperative prophylactic intravesical chemotherapy to prevent tumor cell implantation. Two prospective randomized clinical trials confirmed that early single intravesical instillation of pirarubicin or mitomycin C could reduce bladder recurrence after RNU [22, 23]. Furthermore, Long et al. demonstrated that patients with positive VUC seemed to be more sensitive to intravesical chemotherapy [24]. Therefore, we hypothesized that positive VUC could guide risk stratification to select suitable patients for intravesical chemotherapy and formulate the appropriate frequency of cystoscopies during follow-up.

Along with cystoscopy, preoperative VUC is advocated as the standard method for the diagnosis and surveillance of bladder cancer [25]. Moreover, other studies showed that positive preoperative VUC is associated with disease recurrence and cancer-specific mortality after transurethral resection of bladder tumors [26–28]. The loss of intercellular adhesion is one of the critical biological processes for cancer cells acquiring invasive and metastatic potential, and thus positive VUC could indicate the fragility of intercellular adhesion and reflect the aggressiveness of bladder cancer [26]. Similarly, Sakano et al. found that preoperative VUC increased cancer-specific mortality in UTUC patients [10], but 116 patients (22%) synchronously suffered bladder cancer, which made it difficult to identify the source of malignant cells in urine.

After excluding patients experiencing bladder cancer before and/or during RNU, the present study proved that preoperative VUC independently not only increased the risk of cancer-specific mortality (HR = 1.87, 95% CI 1.10–3.18; *P* = 0.020) but also the risk of disease recurrence (HR = 1.80, 95% CI 1.08–2.99; *P* = 0.023). The sensitivity of VUC for detecting high-grade and invasive bladder tumors was up to 84% but was only 16% for low-grade tumors [29]. In a study to evaluate the predictive value of urine cytology for worse pathological outcomes of UTUC, Chen et al. reported positive urine cytology was associated with high-grade disease [11]. In other studies, preoperative VUC also has been explored as a predictive tool for high-grade muscle-invasive (pT2–pT4) and/or non-organ-confined (pT3 or greater, or lymph node metastasis) UTUC [12, 13]. Sakano et al. did not further explore the relationship between positive VUC and the aggressive features of UTUC [10]. Our data showed that preoperative VUC was significantly associated with high-grade UTUC (OR = 2.23, 95% CI 1.15–4.52), but had no interaction with tumor grade in survival analyses. Due to its ability to reflect the fragility of intercellular adhesion and its relationship with invasive disease, positive preoperative VUC might be an independent prognosticator in predicting disease recurrence and cancer-specific death in UTUC.

In accordance with previous studies, we found that pathologic stage and advanced age were independent predictors of disease recurrence and survival in UTUC. LVI poorly affected disease recurrence (OR = 2.66, 95% CI 1.32–5.34) and overall death (OR = 2.22, 95% CI 1.14–4.33) in pTa-4N0M0 patients. In a large multicenter study of > 1400 patients, Kikuchi et al. found that LVI was an independent predictor of clinical outcomes in node-negative UTUC patients after RNU [30]. LVI seemed to help identify a subgroup of patients with micro-metastases or false-negative lymph node status.

The present study has several limitations. First, this was a single-center retrospective design with a limited number of patients, which carried an intrinsic bias. Preoperative VUC was not a prognosticator for OS. The possible explanation was that the overall follow-up period was too restricted to observe the positive impact of VUC on OS. Second, patients who did not retain VUC were not included in the present analysis. Third, information of postoperative intravenous chemotherapy was not included in this study. Based on the National Cancer Database, Seisen et al. reported an OS benefit in pT3/T4 and/or pN+ UTUC patients who received adjuvant chemotherapy [31]. However, there are insufficient data on which to base a recommendation of systemic chemotherapy for UTUC [1]. Fourth, the EAU guideline considers neutrophil-to-lymphocyte ratio (NLR) as a prognostic factor for UTUC patients, which is an easily measurable and reproducible marker of the systemic immune response [1]. Several biomarkers, such as C-reactive protein, platelet counts, and white blood cell counts, have also been found to represent prognostic factors in various human cancer types. However, the laboratory data were unavailable in the present study. Thus, we did not explore the prognostic impact of these biomarkers on UTUC.

## Conclusions

In conclusion, we found that preoperative VUC is an independent prognostic factor for intravesical recurrence after RNU for UTUC, and a strong supportive mechanism is intraluminal seeding and implantation of cancer cells from the upper urinary tract to the bladder. Furthermore, preoperative VUC significantly increases the risk of disease recurrence and cancer-specific death for UTUC patients, but not overall death. Positive VUC may be associated with the fragility of intercellular adhesion and the high grade of UTUC.

## Data Availability

The datasets used and/or analyzed during the current study are available from the corresponding author upon reasonable request.

## Abbreviations

UTUC: Upper urinary tract urothelial carcinoma
RNU: Radical nephroureterectomy
CSS: Cancer-specific survival
VUC: Voided urine cytology
CT: Computed tomography
LVI: Lymphovascular invasion
RFS: Recurrence-free survival
OS: Overall survival
HR: Hazard ratio
CI: Confidence interval
URS: Ureteroscopy
